# Supplementary material for: More than magnetic isolation: Dynabeads as strong Raman reporters towards simultaneous capture and identification of targets
Source: ArXiv. 2023 Jul 22:arXiv:2305.07199v2. Originally published 2023 May 12. Preprint. [Version 2] (PMC10197730)
Supplement: 1 [file NIHPP2305.07199V2-supplement-1.pdf]

Supplementary Information

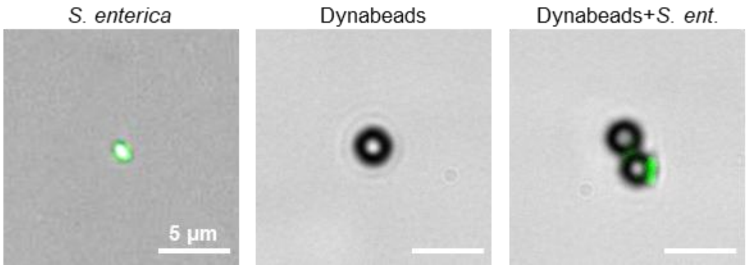

Figure S1. Fluorescence microscopy of *S. enterica* (left), Dynabeads (middle), and *S. enterica*-bound Dynabeads (right) showing size, shape, and tight binding interaction. All scale bars are 5 μm.

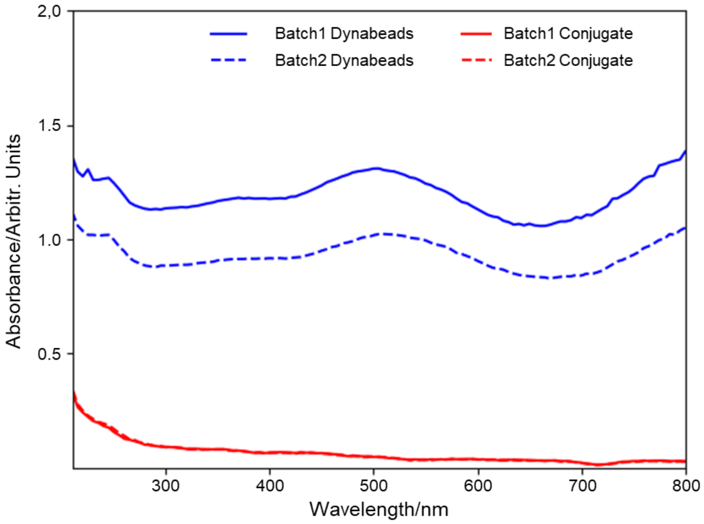

Figure S2. UV-Vis spectra of two batches of Dynabeads and *S. enterica*-bound Dynabeads showing consistent absorbance trends across batches. Decreases in absorbance from Batch 2 could be due to the degradation of Dynabeads' as they decay past their shelf life.

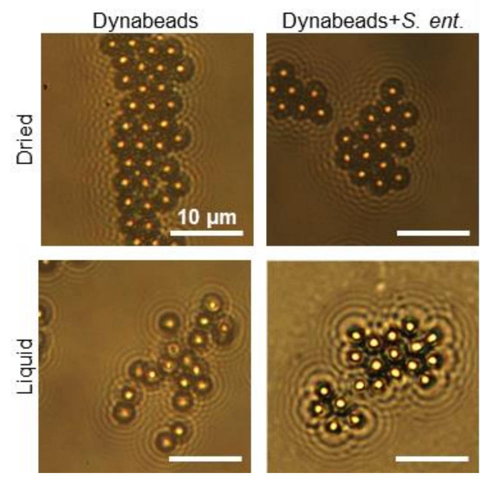

Figure S3. Clustering of Dynabeads in dried and liquid samples before and after conjugation to *S. enterica*. All scale bars are 10 μm.

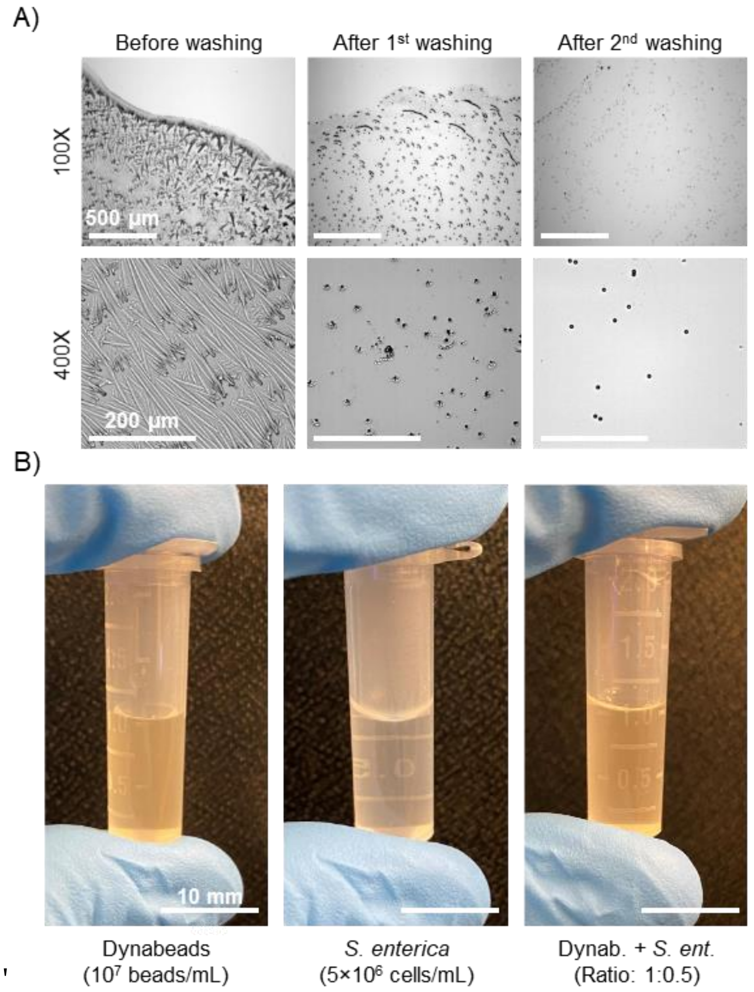

Figure S4. Preparation of samples for Raman interrogation. (A) Optical images of dried Dynabeads with 0, 1, and 2 washing cycles. Most residues in the stock buffer (sodium azide and BSA) can be removed after two cycles of washing. (B) Dynabeads and *S. enterica* samples were

prepared at a fixed concentration of 10^7 beads/mL and 5×10^6 cells/mL respectively. For conjugation, a ratio of 1:0.5 Dynabeads to *S. enterica* was used.

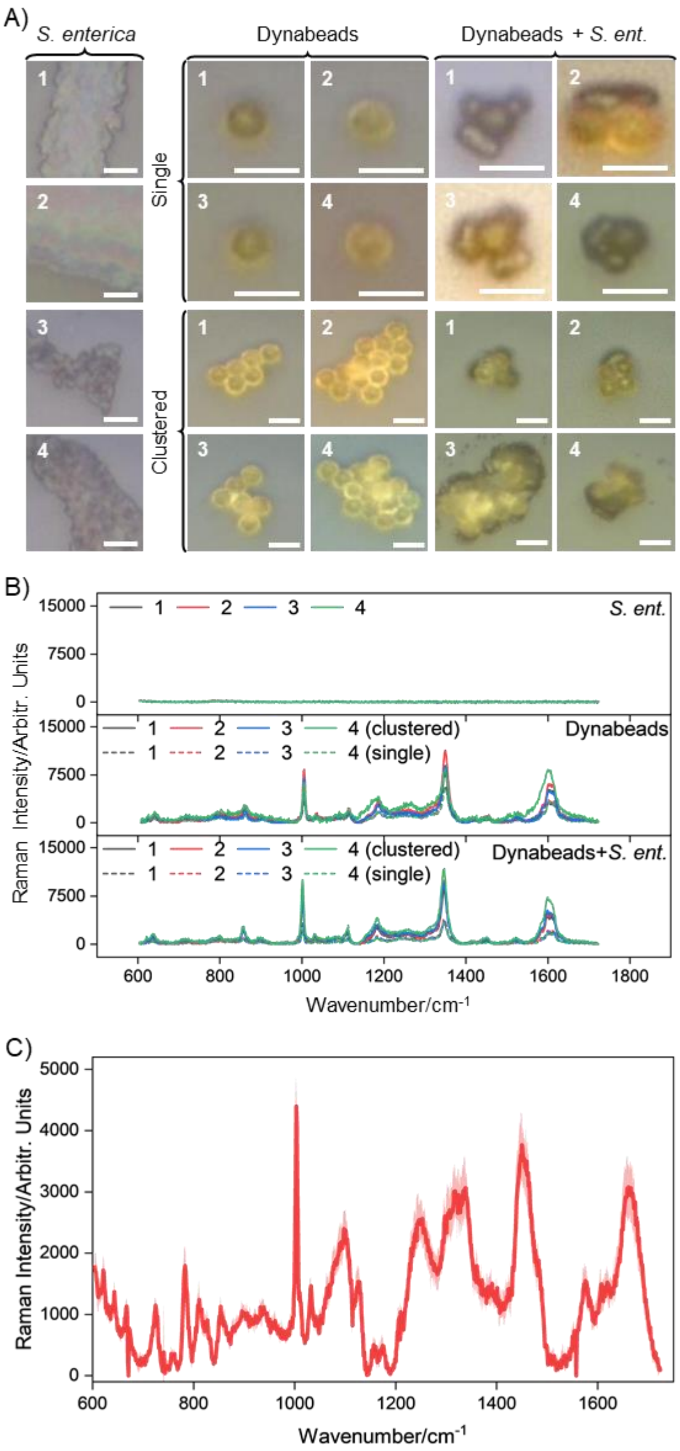

Figure S5. Raman spectra from dried samples. (A) Brightfield images of the four different sample locations where spectral data was collected (scale bar = 5 μm). (B) Raman spectra from *S. enterica* (top), single- and clustered Dynabeads (middle), and single- and clustered *S. enterica*-bound Dynabeads. (C) Raman spectra of *S. enterica* with modified acquisition settings for a

higher resolution ($C_{S. ent}$= 3×10^9/mL, laser power= 100 mW, acquisition= 60 s). Signature peaks can be seen at 1000, 1350, 1450, and 1660 cm^{-1}, similar to previous literature reports.

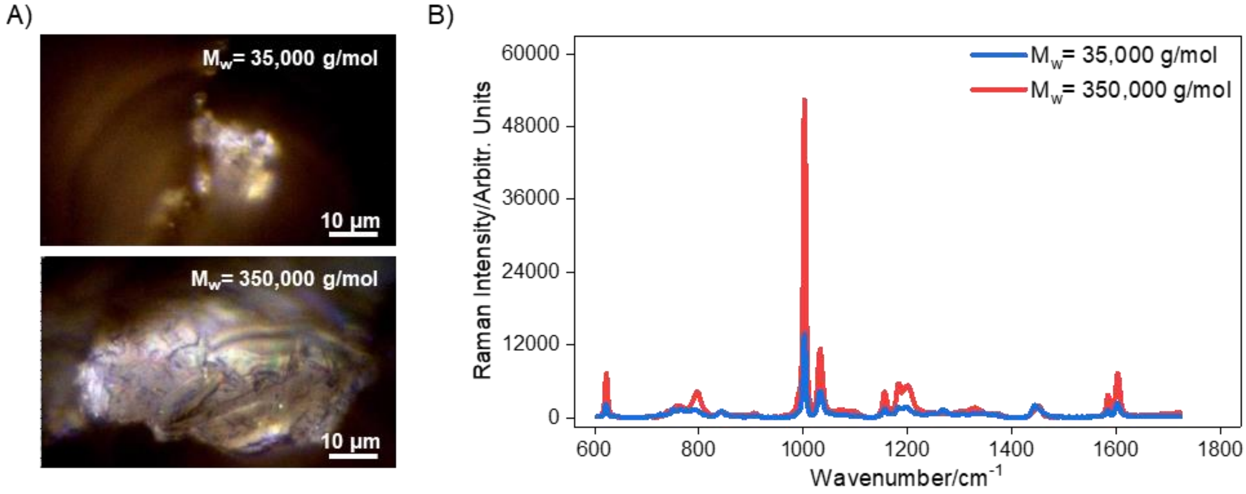

Figure S6. Raman spectra from polystyrene (PS). (A) Brightfield images of PS with different molecular weights (M_w): 35,000 and 350,000 g/mol. Images show where the incident laser was focused for Raman interrogation. (B) Raman spectra of PS of different M_w. Prominent peaks at 1000 and 1600 match observation in Dynabeads-containing samples, indicating large contributions of polystyrene to Raman spectra of Dynabeads.

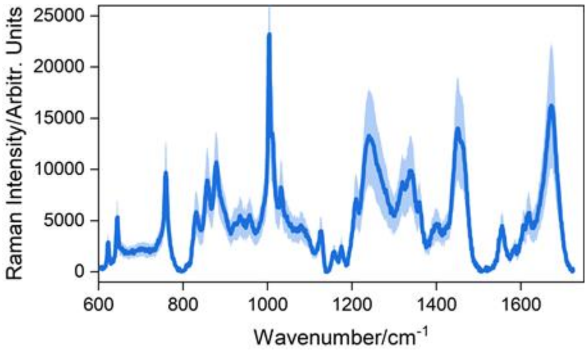

Figure S7. Raman spectra of anti-*Salmonella* antibodies with 1 min acquisition at 100 mW. Major peaks appear at 1004, 1243, 1452, and 1674 cm^{-1}.

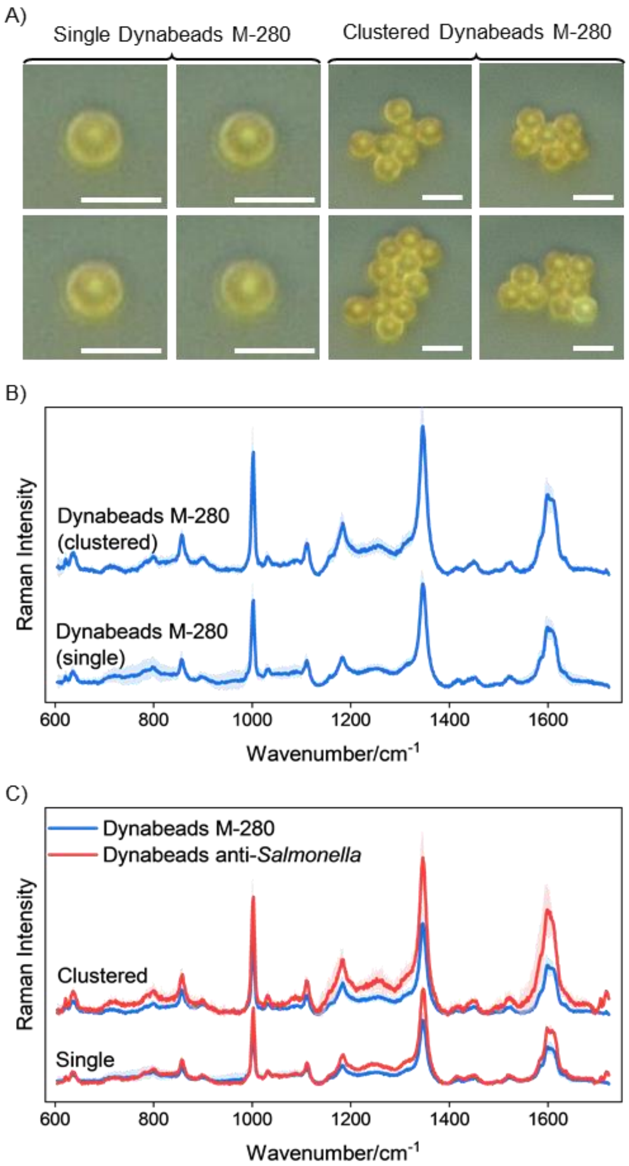

Figure S8. Raman spectra of Dynabeads M-280 Tosylactivated, which are surface-activated Dynabeads lacking surface antibodies. (A) Brightfield images of clustered and single Dynabeads M-280 Tosylactivated (Scale bar: 5 µm). (B) Raman spectra of single and clustered Dynabeads M-280 Tosylactivated showing strong peaks around 1000, 1350, and 1600 cm^{-1}. (C) Overlap of Raman spectra from Dynbeads M-280 Tosylactivated and Dynabeads anti-*Salmonella*, showing a direct match of Raman signal and indicating little to no contribution from surface antibodies on Dynabeads anti-*Salmonella*.

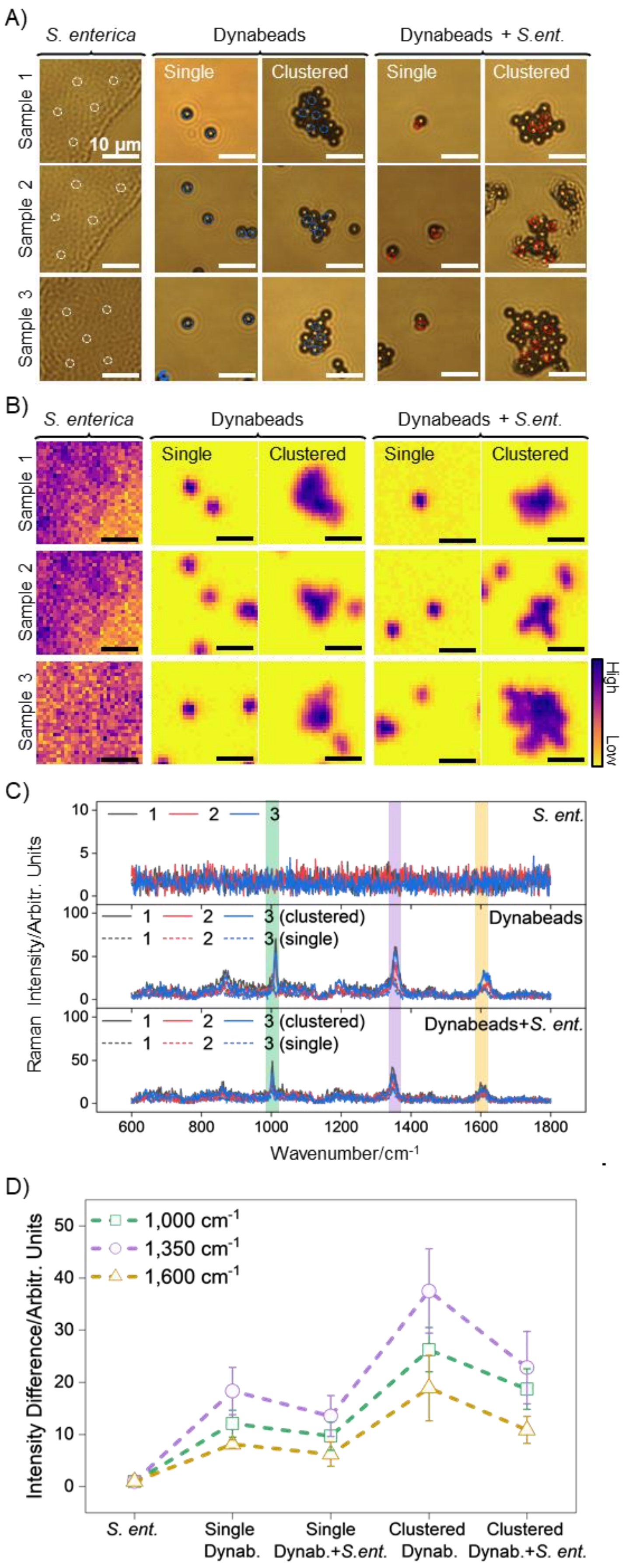

A)
S. enterica
Dynabeads
Dynabeads + S.ent.
Single
Clustered
Single
Clustered
Sample 1
Sample 2
Sample 3
10 µm
B)
S. enterica
Dynabeads
Dynabeads + S.ent.
Single
Clustered
Single
Clustered
High
Low
C)
1 2 3
S. ent.
1 2 3 (clustered)
1 2 3 (single)
Dynabeads
Dynabeads+S. ent.
Raman Intensity/Arbitr. Units
Wavenumber/cm-1
D)
1,000 cm-1
1,350 cm-1
1,600 cm-1
Intensity Difference/Arbitr. Units
S. ent.
Single Dynab.
Single Dynab.+S.ent.
Clustered Dynab.
Clustered Dynab.+S. ent.

Figure S9. Raman map from dried samples with 785 nm laser under 0.5 s exposure and 7.5 mW power. (A) Data from three different locations were collected and analyzed (scale bar = 10 μm). Highly concentrated pellets were selected as *S. enterica* region. Locations with visible *S. enterica* and Dynabeads in the form of single and clustered formats, are selected as *S. enterica*-bound Dynabeads region. (B) Intensity maps after combining intensities at selected wavenumbers; 1,000, 1,350, and 1,600 cm^{-1}. At this acquisition parameter no detectable signature is observed from *S. enterica* only sample, mainly background intensity is detected. (C) Raman spectra from *S. enterica* don't show specific signature peaks under the acquisition parameters. In contrast, Dynabeads alone and *S. enterica*-bound Dynabeads show strong signature peaks from locations circled in dotted lines in the bright field images in A with notable peaks at 1000, 1350, and 1600 cm^{-1}. (D) Intensity difference factors as compared to the bacteria only baseline shows a similar trend as the single point measurement in Figure 2 with overall higher intensity recorded from clustered Dynabeads.

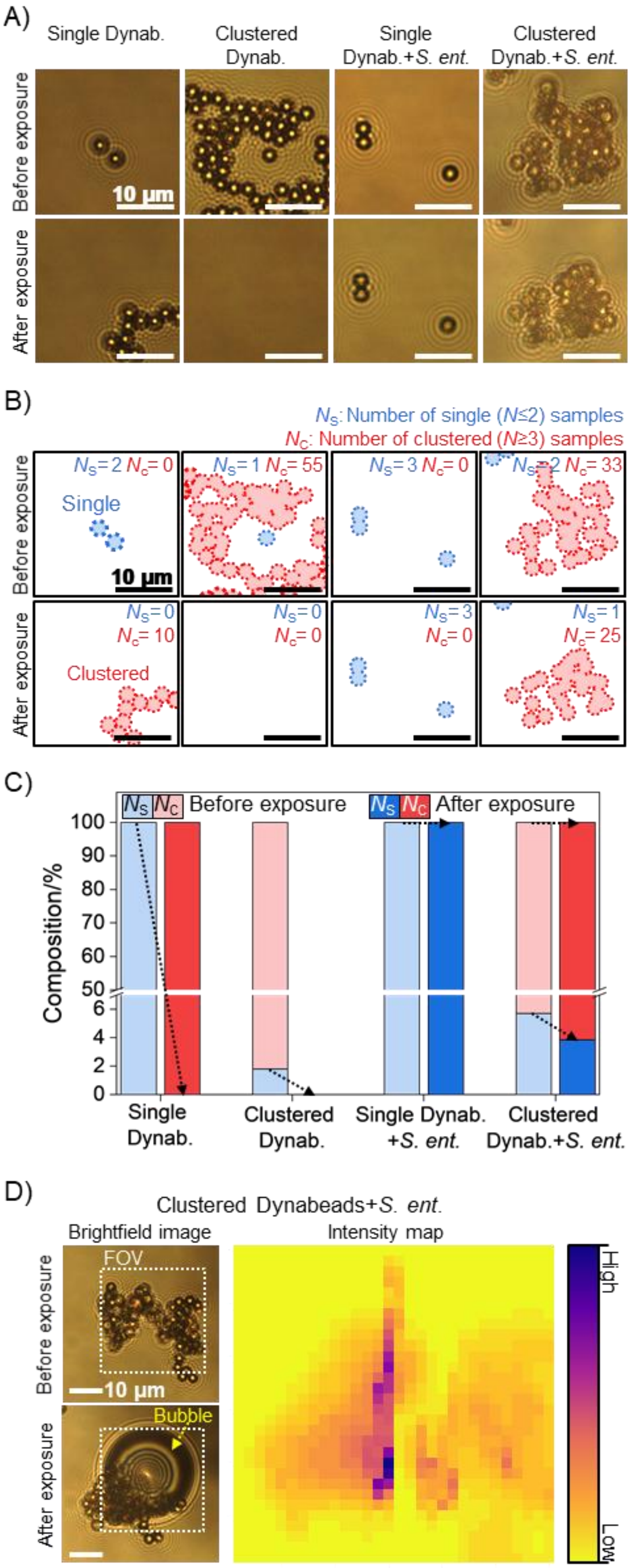

Figure S10. Dislocation of Dynabeads and *S. enterica*-bound Dynabeads before/after exposure to 75 mW-powered laser (λ= 785 nm) for 0.5 s. (A) Each sample was observed using a

brightfield microscope, top showing before and bottom after exposure to laser. (B) Classification of samples in FOV, as single (blue circles, $N \leq 2$) and clustered (red circles, $N \geq 3$) forms, showing distinct differences in Dynabeads and *S. enterica*-bound Dynabeads; all of Dynabeads were dislocated, but none- to few *S. enterica*-bound Dynabeads did. (C) Quantitative analysis on the composition of the number of single (N_S) and clustered (N_C) samples. For Dynabeads, all samples were entirely dislocated; two single Dynabeads were observed in FOV for single bead measurement before laser exposure but they were dislocated and replaced by a cluster after exposure. In the case of single bead measurement, pre-exposure, one single Dynabead (1.2 % composition) and a cluster of 55 Dynabeads (98.2 %) were observed. Post-exposure, all of the Dynabeads were dislocated from FOV, resulting in 0 % of each composition. For single, *S. enterica*-bound Dynabeads, three single samples (100 % in the composition of the singles) were observed, and they retained their position post laser exposure. For clustered *S. enterica*-bound Dynabeads, two singles (5.7 % in composition)- and cluster of 33 (94.3 %) were observed pre-exposure, and one single (3.8 %)- and cluster of 25 (96.2 %) were observed post- exposure. (D) Generation of bubbles inside clustered *S. enterica*-bound Dynabeads induced by the laser exposure.

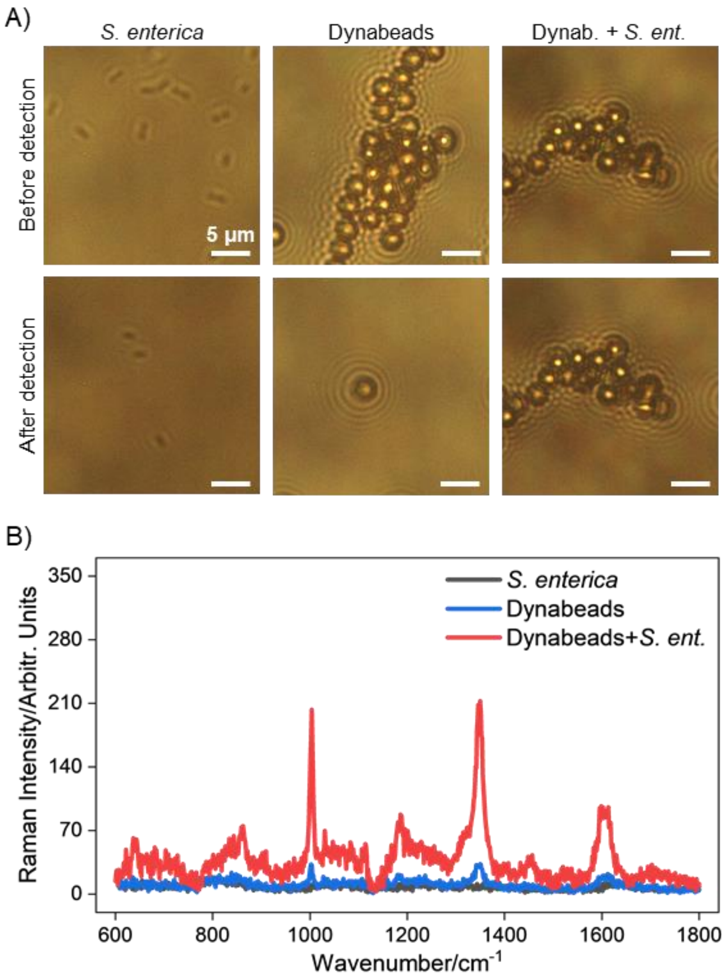

Figure S11. Effects of laser exposure on sample dislocation in liquid. (A) Brightfield images of each sample before- and after laser exposure during Raman interrogation. Only *S. enterica*-bound Dynabeads remain in place post-exposure. (B) Raman spectra from each sample at 75 mW-powered laser (λ= 785 nm) for 0.5 s. The spectra from *S. enterica*-bound Dynabeads showed the highest intensities compared to other samples.

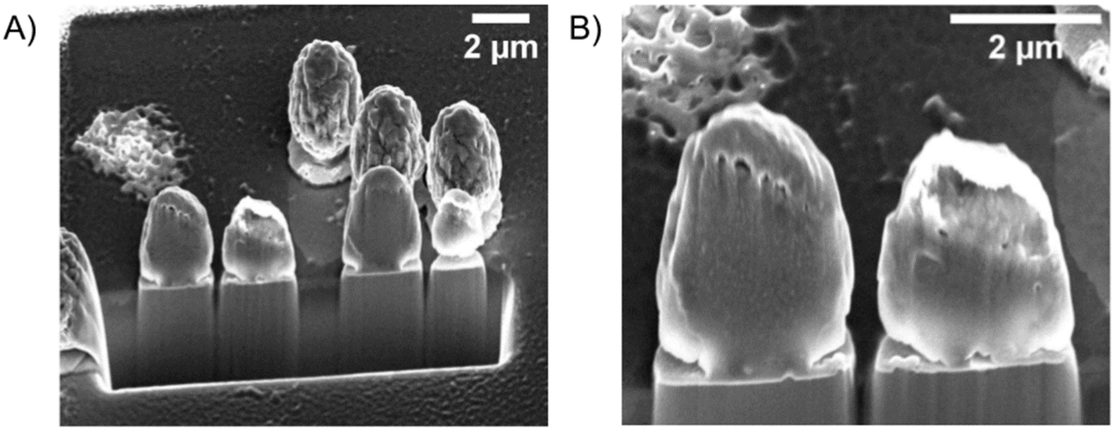

Figure S12. FIB analysis of Dynabeads. (A) Cross sectional image of 4 beads compared to 3 uncut beads also in the field of view. (B) Close up image of cross sectional view of Dynabeads exposes amorphous structure with no specific physical pattern of iron oxide core particles except for faint white dots.

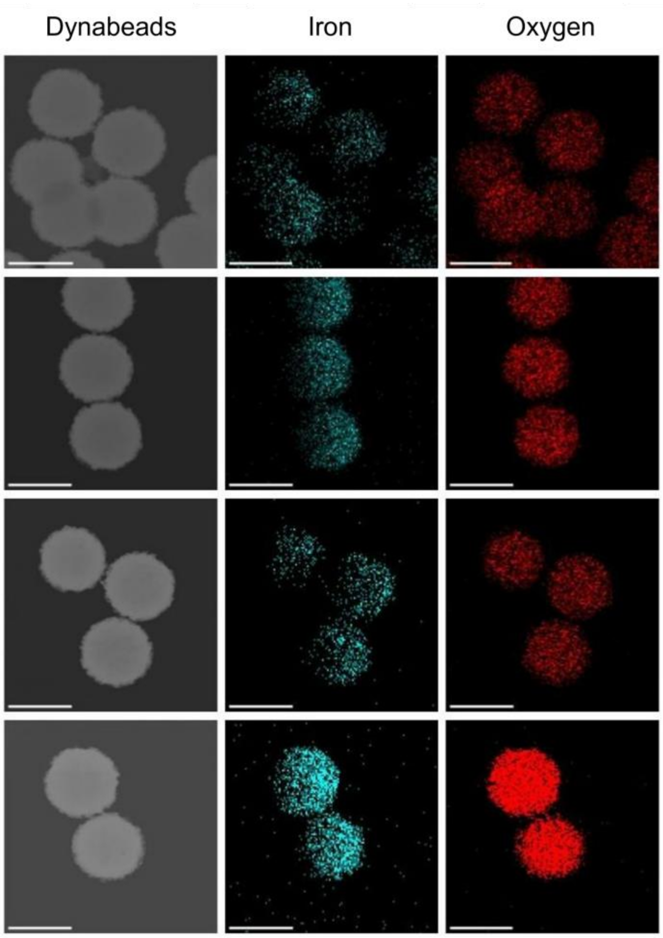

Figure S13. EDX images of Dynabeads (left) with corresponding regions of iron (middle) and oxygen (right). Oxygen distribution is uniform throughout the core, but iron distribution favors one side of the bead. All scale bars are 2 μm.

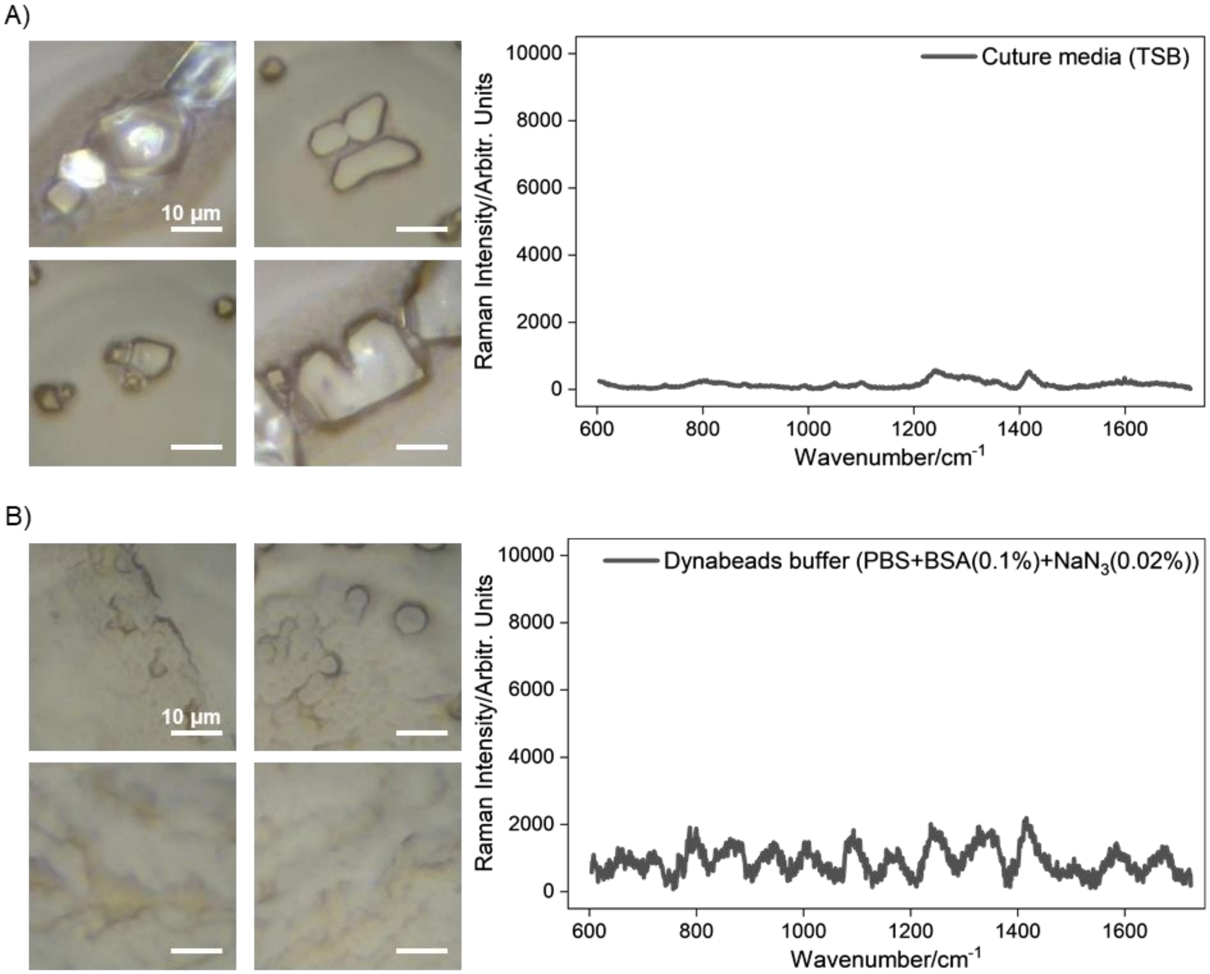

Figure S14. Raman interrogation of buffer solutions. (A) Brightfield images and Raman spectra from dried cell culture media (TSB). (B) Brightfield images and Raman spectra from Dynabead stock buffer (PBS, BSA, sodium azide). Both A and B demonstrate that the observed Raman signal of Dynabeads is not coming from residual buffer solution.

Table S1. Experimental conditions of Raman spectroscopy systems used in this study.

System	Detection method	Target sample	Sample condition	Laser configuration				
				λ/nm	Spot size /μm	Power /mW	Exposure time/s	Accumul-ation
Renishaw Invia Reflex	Single point	Dynabeads anti-*Salmonella*, *S. enterica*, anti-*Salmonella* antibody (CSA-1), polystyrene (M_w= 35000, 350000 g/mol), Dynabeads buffer, TSB	Dried	785	1	10	1	30
		Dynabeads M-280		785	1	5	1	30
		Anti-*Salmonella* antibody (CSA-1),		785	1	100	2	30
Customized	Imaging mode (30×30 μm)	Dynabeads anti-*Salmonella*, *S. enterica*	Dried	785	1	7	0.5	1
			Liquid	785	1	75	0.5	1
