# Supplementary material for: More than magnetic isolation: Dynabeads as strong Raman reporters towards simultaneous capture and identification of targets
Source: ArXiv. 2023 May 12:arXiv:2305.07199v1. Preprint. [Version 1] (PMC10197730)
Supplement: Supplementary file 1 [file NIHPP2305.07199v1-supplement-1.pdf]

Supplementary Information

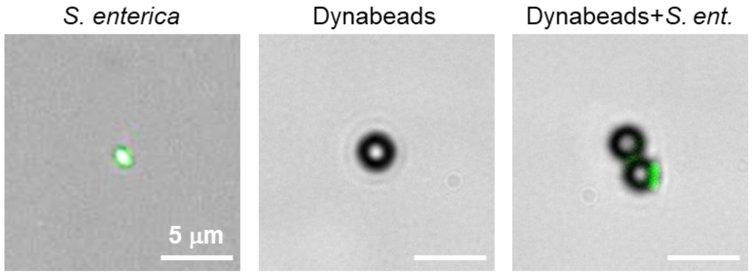

Figure S1. Fluorescence microscopy of *S. enterica* (left), Dynabeads (middle), and *S. enterica*-bound Dynabeads (right) showing size, shape, and tight binding interaction. All scale bars are 5 μm.

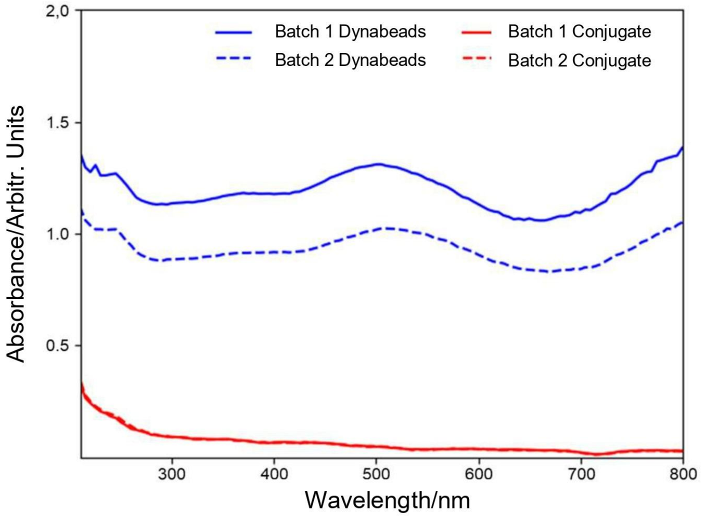

Figure S2. UV-Vis spectra of two batches of Dynabeads and *S. enterica*-bound Dynabeads showing consistent absorbance trends across batches. Decreases in absorbance from Batch 2 could be due to the degradation of Dynabeads' as they decay past their shelf life.

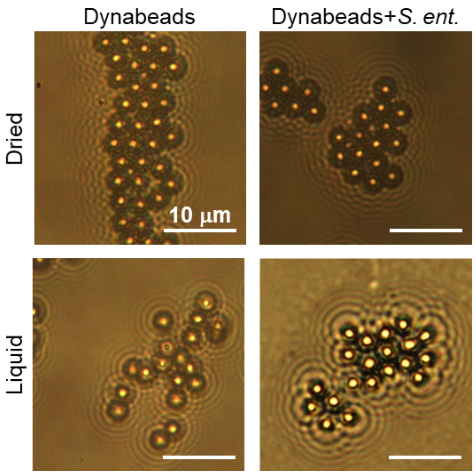

Figure S3. Clustering of Dynabeads in dried and liquid samples before and after conjugation to *S. enterica*. All scale bars are 10 µm.

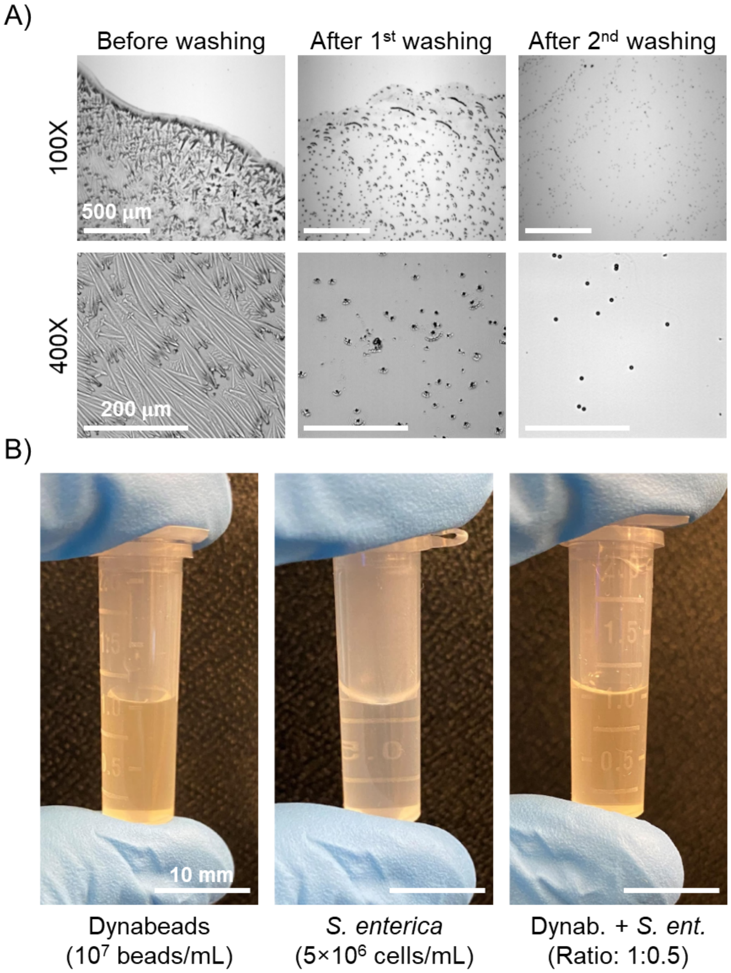

Figure S4. Preparation of samples for Raman interrogation. (A) Optical images of dried Dynabeads with 0, 1, and 2 washing cycles. Most residues in the stock buffer (sodium azide and BSA) can be removed after two cycles of washing. (B) Dynabeads and *S. enterica* samples were prepared at fixed concentration of 10^7 beads/mL and 5×10^6 cells/mL respectively. For conjugation, a ratio of 1:0.5 Dynabeads to *S. enterica* was used.

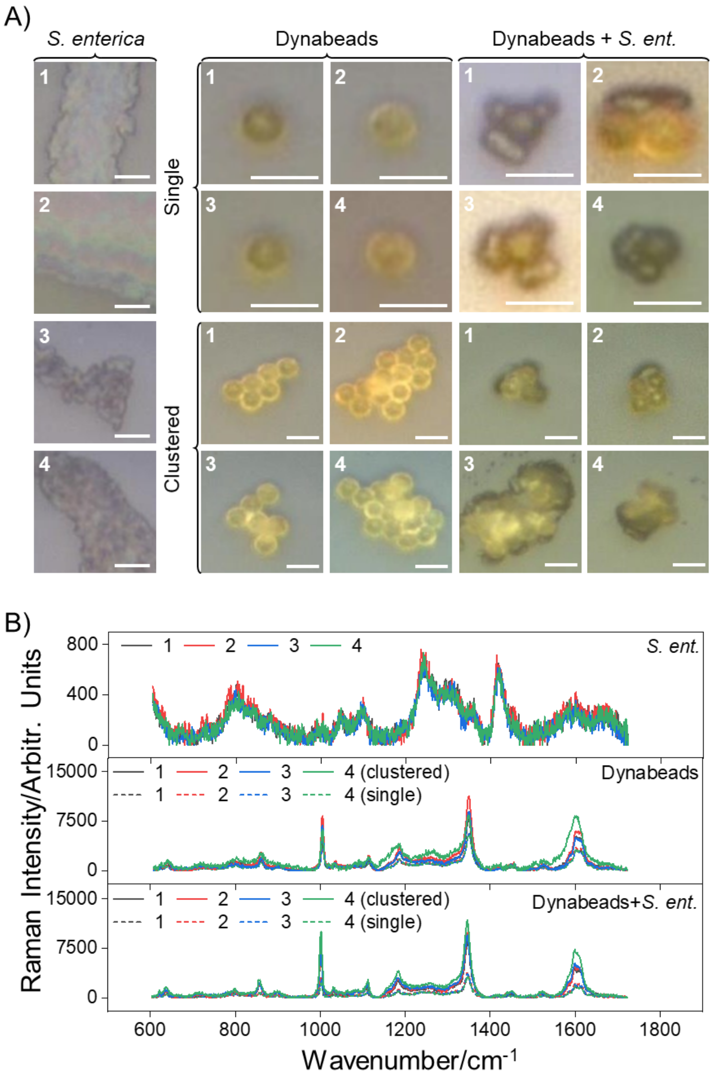

Figure S5. Raman spectra from dried samples. (A) Data from four different sample locations were collected and analyzed (scale bar = 5 μm). (B) *S. enterica* (top), single- and clustered Dynabeads (middle), and single- and clustered *S. enterica*-bound Dynabeads. Signal from clustered beads is overall larger than that of single beads in both conditions.

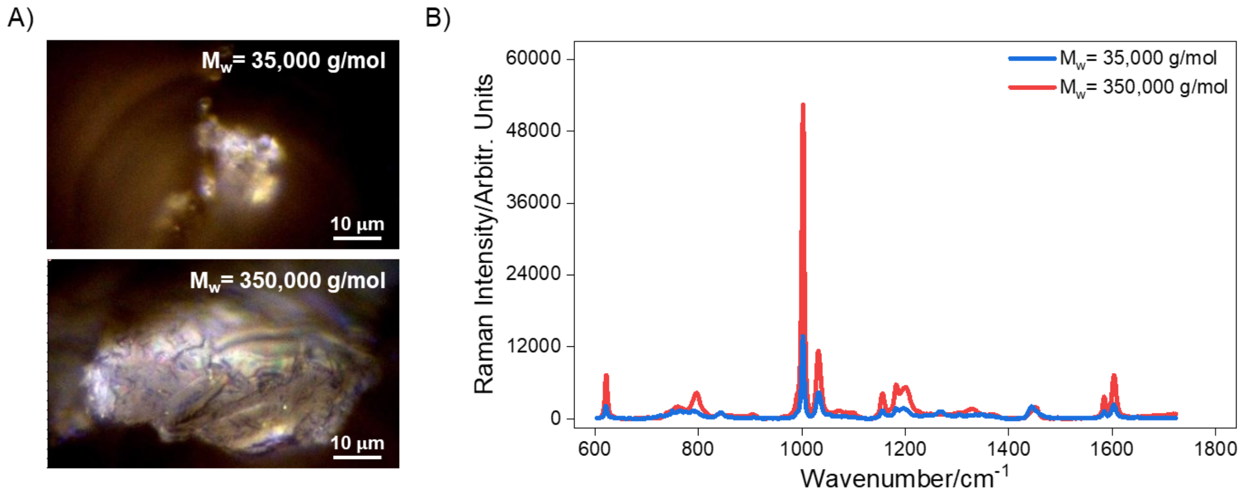

Figure S6. Raman spectra from polystyrene (PS). (A) Brightfield images of PS with different molecular weights (M_w): 35,000 and 350,000 g/mol. Images show where the incident laser was focused for Raman interrogation. (B) Raman spectra of PS of different M_w. Prominent peaks at 1000 and 1600 match observation in Dynabeads-containing samples, indicating large contributions of polystyrene to Raman spectra of Dynabeads.

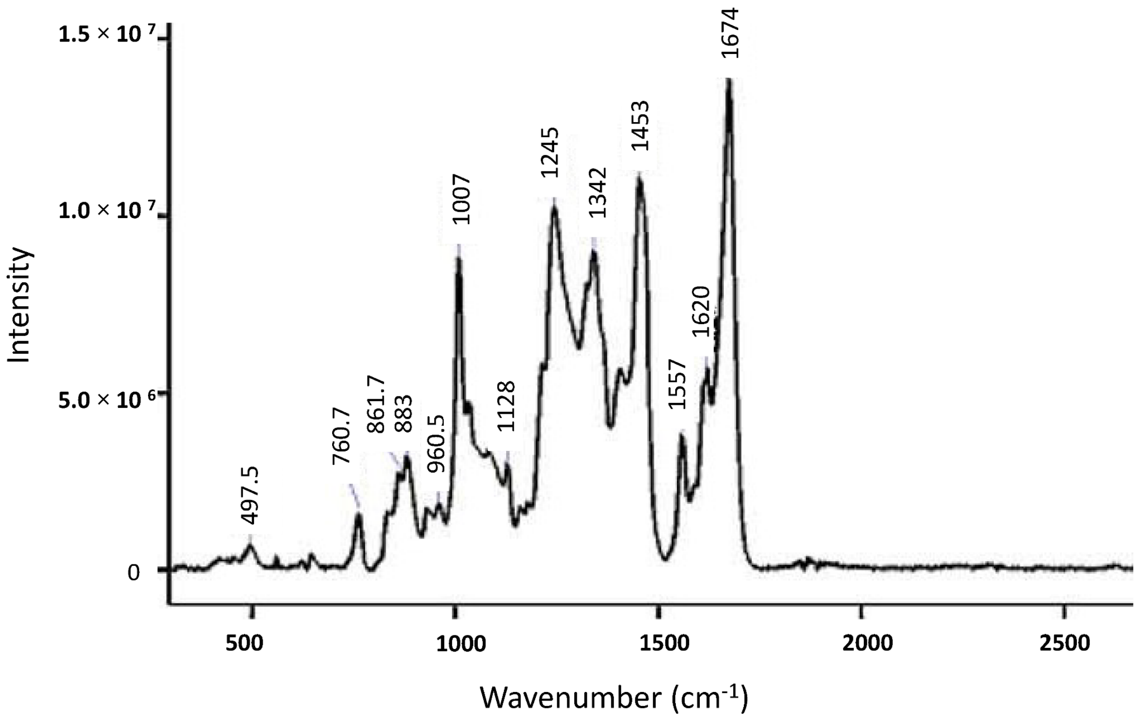

Figure S7. Raman spectra of typical monoclonal antibodies like that of anti-*Salmonella*. Special attention is given to peaks around 1350, and 1600 marking amide, α-helix and □-sheet structure as seen in the observed spectra of Dynabeads. Reproduced with permission fromWen, Z.-Q.[1] Copyright Elsevier (2007).

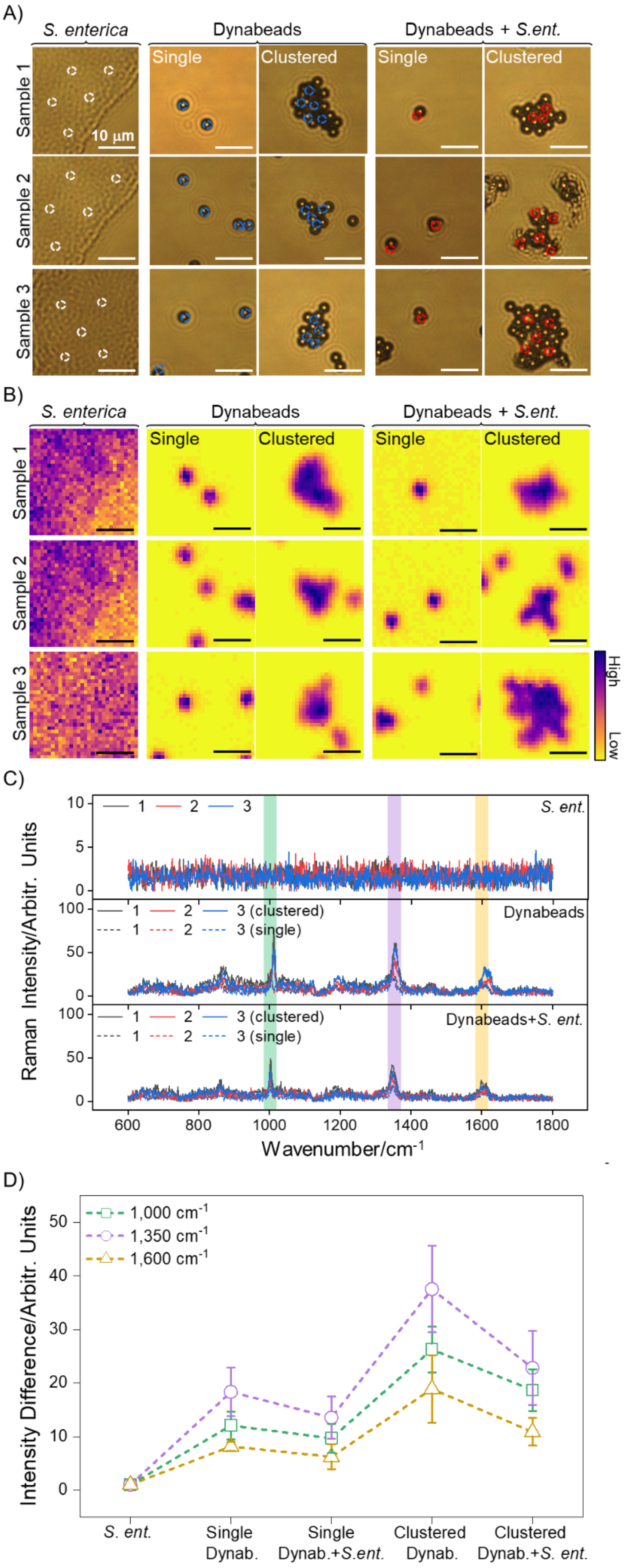
A)
S. enterica
Dynabeads
Dynabeads + S.ent.
Single
Clustered
Single
Clustered
Sample 1
Sample 2
Sample 3
10 µm
B)
S. enterica
Dynabeads
Dynabeads + S.ent.
Single
Clustered
Single
Clustered
Sample 1
Sample 2
Sample 3
High
Low
C)
1 2 3
S. ent.
1 2 3 (clustered)
1 2 3 (single)
Dynabeads
Dynabeads+S. ent.
Raman Intensity/Arbitr. Units
Wavenumber/cm-1
D)
1,000 cm-1
1,350 cm-1
1,600 cm-1
Intensity Difference/Arbitr. Units
S. ent.
Single Dynab.
Single Dynab.+S.ent.
Clustered Dynab.
Clustered Dynab.+S. ent.

Figure S8. Raman map from dried samples. (A) Data from three different locations were collected and analyzed (scale bar = 10 μm). Highly concentrated pellets were selected as *S. enterica* region. Locations with visible *S. enterica* and Dynabeads in the form of single and clustered formats, are selected as *S. enterica*-bound Dynabeads region. (B) Intensity maps after combining intensities at selected wavenumbers; 1,000, 1,350, and 1,600 cm^{-1}. At this acquisition parameter no detectable signature is observed from *S. enterica* only sample, mainly background intensity is detected. (C) Raman spectra from *S. enterica* don't show specific signature peaks under the 0.5 sec exposure 7.5 mW-power laser acquisition parameters. In contrast, Dynabeads alone and *S. enterica*-bound Dynabeads show strong signature peaks from locations circled in dotted lines in the bright field images in A with notable peaks at 1000, 1350, and 1600 cm^{-1}. (D) Intensity difference factors as compared to the bacteria only baseline shows a similar trend as the single point measurement in Figure 2; with overall higher intensity recorded from clustered Dynabeads.

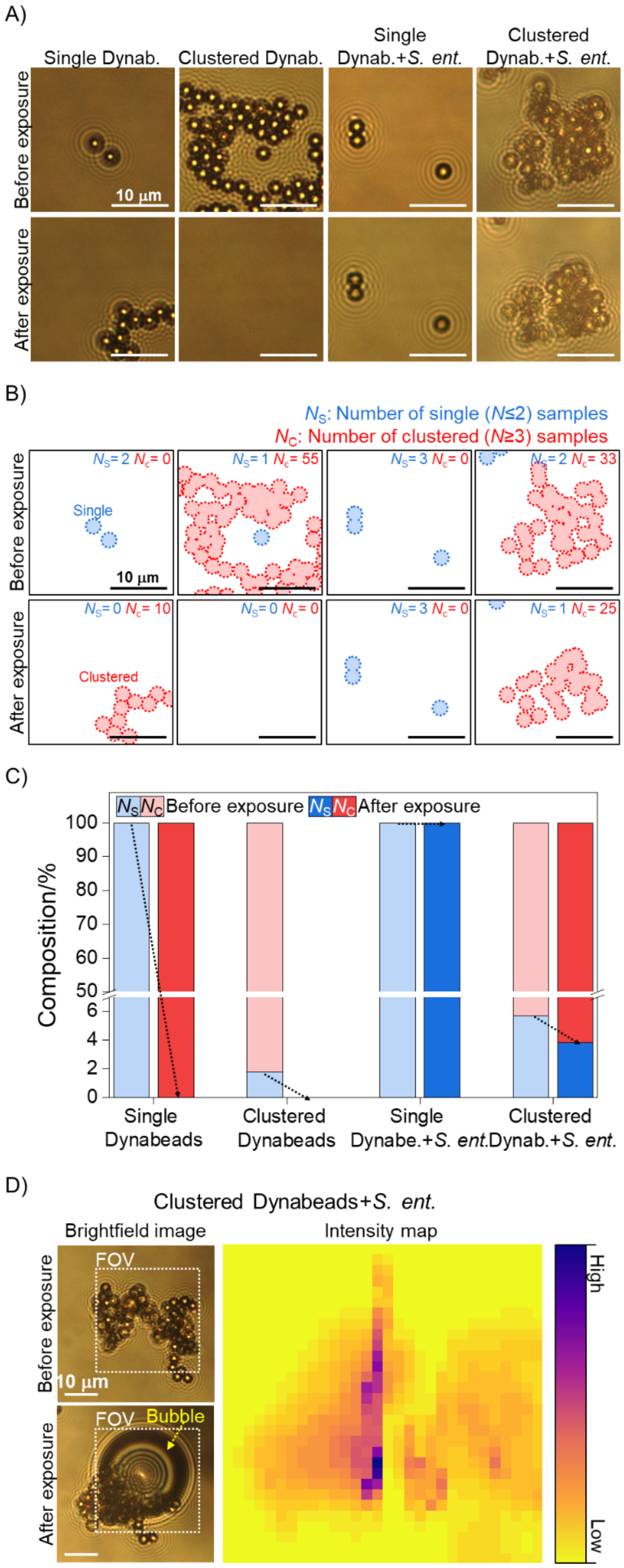

A)
Single Dynab.
Clustered Dynab.
Single Dynab.+S. ent.
Clustered Dynab.+S. ent.
Before exposure
After exposure
10 μm
B)
N_S: Number of single (N≤2) samples
N_C: Number of clustered (N≥3) samples
N_S= 2 N_c= 0
N_S= 1 N_c= 55
N_S= 3 N_c= 0
N_S= 2 N_c= 33
N_S= 0 N_c= 10
N_S= 0 N_c= 0
N_S= 3 N_c= 0
N_S= 1 N_c= 25
Single
Clustered
C)
N_S N_C Before exposure N_S N_C After exposure
Composition/%
Single Dynabeads
Clustered Dynabeads
Single Dynabe.+S. ent.
Clustered Dynab.+S. ent.
D)
Clustered Dynabeads+S. ent.
Brightfield image
Intensity map
FOV
Bubble
High
Low

Figure S9. Dislocation of Dynabeads and *S. enterica*-bound Dynabeads before/after exposure to 75 mW-powered laser (λ= 785 nm) for 0.5 sec. (A) Each sample was observed using a brightfield microscope, top showing before and bottom after exposure to laser. (B) Classification of samples in FOV, as single (blue circles, $N \leq 2$) and clustered (red circles, $N \geq 3$) forms, showing distinct differences in Dynabeads and *S. enterica*-bound Dynabeads; all of Dynabeads were dislocated, but none- to few *S. enterica*-bound Dynabeads did. (C) Quantitative analysis on the composition of the number of single (N_S) and clustered (N_C) samples. For Dynabeads, all samples were entirely dislocated; Two single Dynabeads were observed in FOV for single bead measurement before laser exposure but they were dislocated and replaced by a cluster after exposure. In the case of single bead measurement, pre-exposure one single Dynabeads (1.2 % composition) and cluster of 55 Dynabeads (98.2 %) were observed, post laser exposure all of Dynabeads were dislocated from FOV, resulting in 0 % of each composition. For single *S. enterica*-bound Dynabeads, three single samples (100 % in the composition of the singles) were observed, and they retained their position post laser exposure as shown in Figure 2. For clustered *S. enterica*-bound Dynabeads, two single (5.7 % in composition)- and cluster of 33 (94.3 %) were observed preexposure, and one single (3.8 %)- and cluster 0f 25 (96.2 %) were observed post exposure. (D) Generation of bubbles inside clustered *S. enterica*-bound Dynabeads induced by the laser exposure.

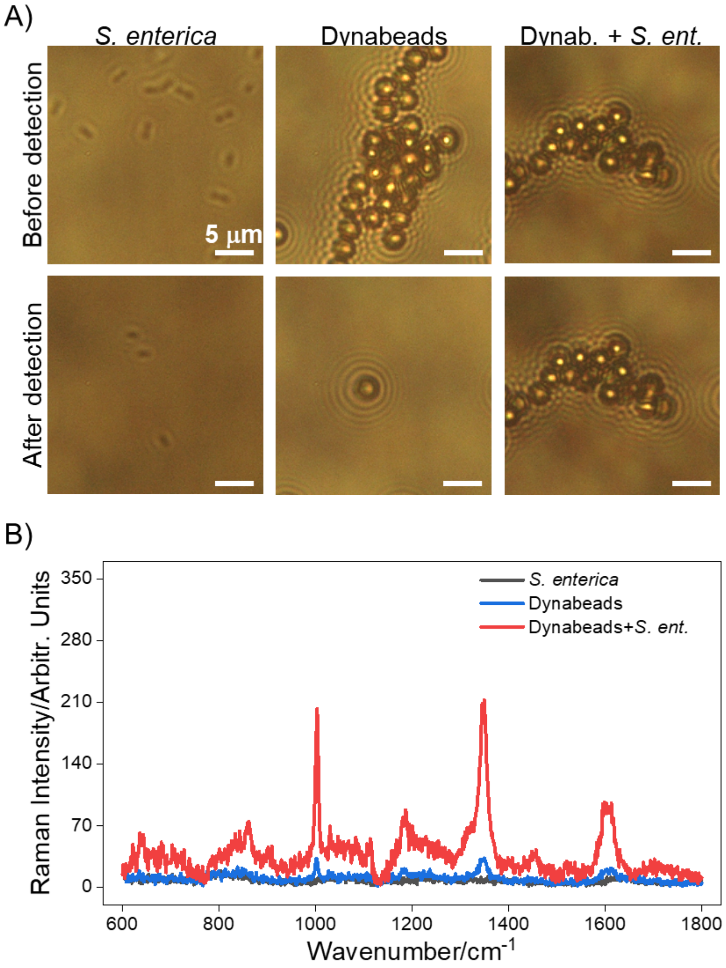

Figure S10. Effects of laser exposure on sample dislocation in liquid. (A) Brightfield images of each sample before- and after laser exposure during Raman interrogation. Only *S. enterica*-bound Dynabeads remain in place post-exposure. (B) Raman spectra from each sample. The spectra from *S. enterica*-bound Dynabeads showed the highest intensities compared to other samples.

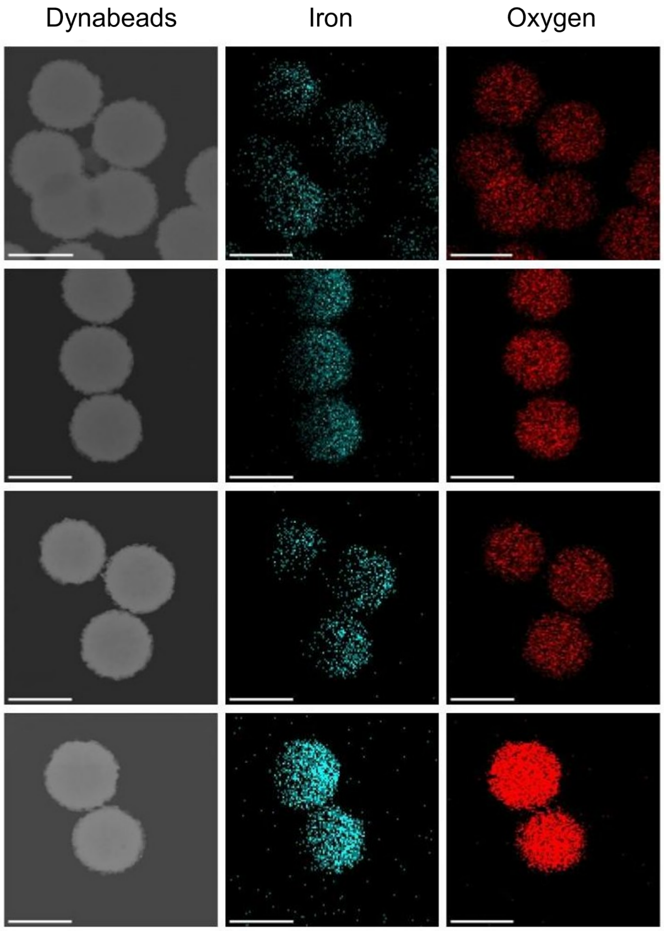

Figure S11. EDX images of Dynabeads (left) with corresponding regions of iron (middle) and oxygen (right). Oxygen distribution is uniform throughout the core, but iron distribution favors one side of the bead. All scale bars are 2 μm.

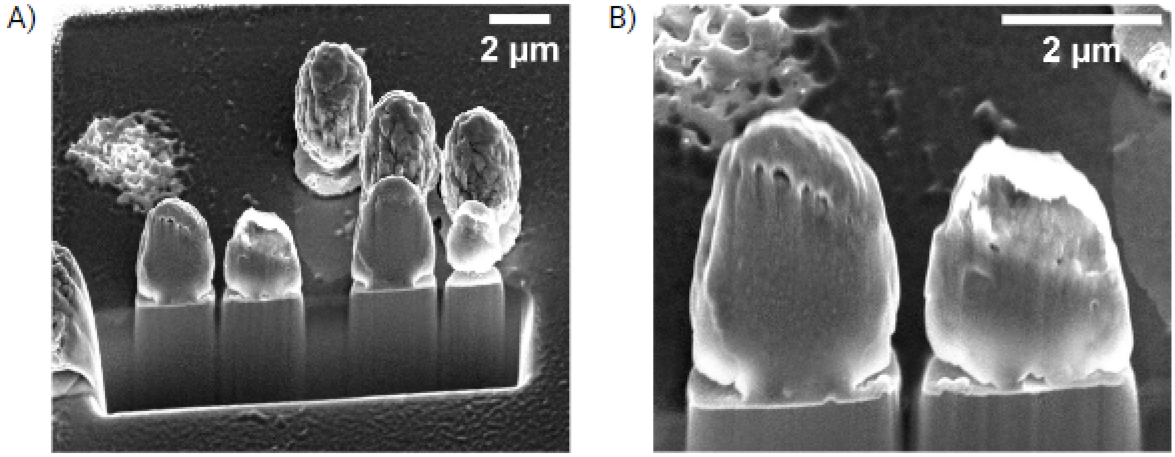

Figure S12. FIB analysis of Dynabeads. (A) Cross sectional image of 4 beads compared to 3 uncut beads also in the field of view. (B) Close up image of cross sectional view of Dynabeads exposes amorphous structure with no specific physical pattern of iron oxide core particles except for faint white dots.

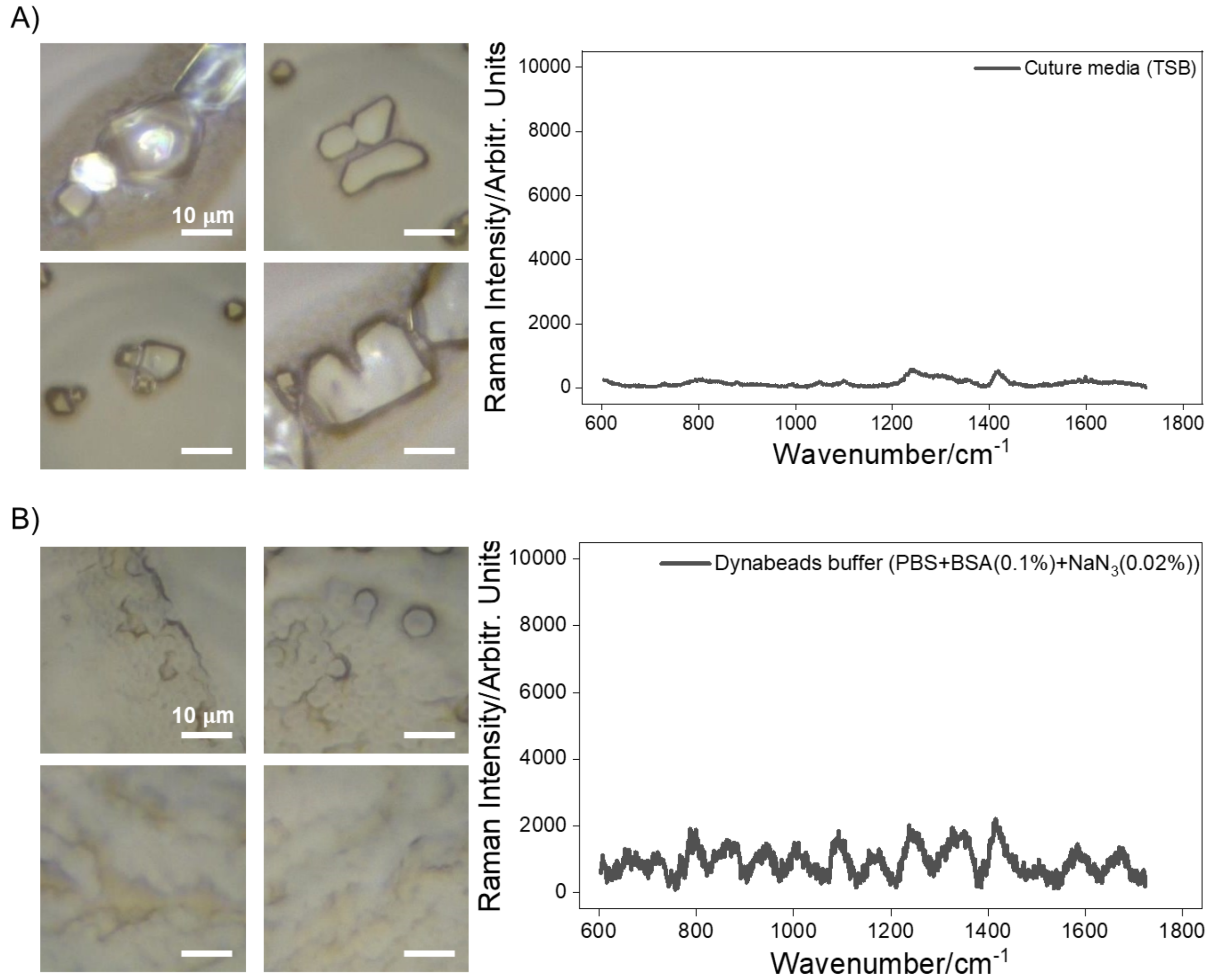

Figure S13. Raman interrogation of buffer solutions. (A) Brightfield images and Raman spectra from dried cell culture media (TSB). (B) Brightfield images and Raman spectra from Dynabead stock buffer (PBS, BSA, sodium azide). Both A and B demonstrate that the observed Raman signal of Dynabeads is not coming from residual buffer solution.

Table S1. Experimental conditions of Raman spectroscopy systems used in this study.

System	Detection method	Sample condition	Laser configuration				
			Wavelength (nm)	Spot size (μm)	Power (mW)	Exposure time (s)	Accumul-ation
Renishaw Invia Reflex	Single point	Dried	785	1	10	1	30
Customized	Imaging mode (30×30 μm)	Dried	785	1	7	0.5	1
		Liquid	785	1	75	0.5	1

References

1. Wen, Z.-Q. Raman spectroscopy of protein pharmaceuticals. *J. Pharm. Sci.* **96**, 2861–2878 (2007)
